# Outcomes after allogeneic hematopoietic stem cell transplantation in acute myeloid leukemia patients with der(1;7)(q10;p10)

**DOI:** 10.1002/jha2.609

**Published:** 2022-11-06

**Authors:** Hiroki Mizumaki, Ken Ishiyama, Jun Aoki, Jinichi Mori, Shohei Mizuno, Noriko Doki, Takahiro Fukuda, Naoyuki Uchida, Masahito Onizuka, Masatsugu Tanaka, Yuta Katayama, Yukiyasu Ozawa, Kazuhiro Ikegame, Satoru Takada, Toshiro Kawakita, Nobuyuki Aotsuka, Yoshiko Atsuta, Masamitsu Yanada

**Affiliations:** ^1^ Department of Hematology Kanazawa University Hospital Kanazawa Japan; ^2^ Department of Hematopoietic Stem Cell Transplantation National Cancer Center Hospital Tokyo Japan; ^3^ Department of Hematology Jyoban Hospital of Tokiwa Foundation Fukushima Japan; ^4^ Division of Hematology Department of Internal Medicine Aichi Medical University Nagakute Japan; ^5^ Hematology Division Tokyo Metropolitan Cancer and Infectious Diseases Center Komagome Hospital Tokyo Japan; ^6^ Department of Hematology Federation of National Public Service Personnel Mutual Aid Associations Toranomon Hospital Tokyo Japan; ^7^ Department of Hematology and Oncology Tokai University School of Medicine Isehara Japan; ^8^ Department of Hematology Kanagawa Cancer Center Yokohama Japan; ^9^ Department of Hematology Hiroshima Red Cross Hospital and Atomic‐bomb Survivors Hospital Hiroshima Japan; ^10^ Department of Hematology Japanese Red Cross Aichi Medical Center Nagoya Daiichi Hospital Nagoya Japan; ^11^ Department of Hematology Hyogo Medical University Hospital Hyogo Japan; ^12^ Leukemia Research Center Saiseikai Maebashi Hospital Maebashi Japan; ^13^ Department of Hematology National Hospital Organization Kumamoto Medical Center Kumamoto Japan; ^14^ Division of Hematology‐Oncology Japanese Red Cross Society Narita Hospital Narita Japan; ^15^ Japanese Data Center for Hematopoietic Cell Transplantation Nagoya Japan; ^16^ Department of Registry Science for Transplant and Cellular Therapy Aichi Medical University School of Medicine Nagakute Japan; ^17^ Department of Hematology and Cell Therapy Aichi Cancer Center Nagoya Japan

**Keywords:** additional chromosomal abnormalities, allogeneic hematopoietic stem cell transplantation, AML, der(1;7)(q10;p10)

## Abstract

The prognosis of acute myeloid leukemia (AML) patients with der(1;7)(q10;p10) who underwent allogeneic hematopoietic stem cell transplantation (allo‐SCT) is unclear due to its rarity. We retrospectively analyzed 151 AML patients with der(1;7)(q10;p10) and compared the findings with those of 853 AML patients with monosomy 7 or chromosome 7q deletion (‐7/del(7q)) using Japanese nationwide registry data. The der(1;7)(q10;p10) group showed significantly better transplant outcomes than the ‐7/del(7q) group. In the multivariate analysis of the der(1;7)(q10;p10) group, additional chromosomal abnormalities and a poor performance status significantly influenced the survival. In conclusion, allo‐SCT is a feasible treatment option for AML patients with der(1;7)(q10;p10).

## 1 INTRODUCTION

Allogeneic hematopoietic stem cell transplantation (allo‐SCT) is a curative therapeutic option for patients with acute myeloid leukemia (AML). Among prognostic factors that influence the outcome of allo‐SCT, cytogenetic abnormalities are the most powerful [1, 2, 3].

Unbalanced whole‐arm translocation between 1p10 and 7q10, typically described as 46, XY (or XX), +1, der(1;7)(q10;p10) (henceforth der(1;7)), is a relatively rare acquired chromosomal abnormality found in myelodysplastic syndrome (MDS) and AML, being reported at frequencies of 1.5%–6% in MDS and 0.2%–2.1% in AML [4]. Some studies have reported unique clinical features of MDS patients with der(1;7) including a strong association with a history of chemoradiotherapy, male predominant, and increased incidence in Asian populations [5, 6, 7, 8].

Recent studies have shown that MDS patients with der(1;7) have a lower incidence of AML transformation and better prognosis than MDS patients with monosomy 7 or partial deletion of the long arm of chromosome 7 (‐7/del(7q)) [4, 5, 8]. However, data regarding the clinical outcome of AML with der(1;7) are lacking.

To clarify the outcomes and prognostic factors in AML patients with der(1;7) who underwent allo‐SCT, we compared transplant outcomes in AML patients with der(1;7) and those with ‐7/del(7q) using nationwide registration data in Japan. We also evaluated the risk factors for transplant outcomes in AML patients with der(1;7).

### 1.1 Patients and methods

Clinical data were provided by the Transplant Registry Unified Management Program (TRUMP) of the Japanese Data Center for Hematopoietic Cell Transplantation (JDCHCT) [9, 10]. The selected patients were ≥16 years old, had AML with der(1;7) or ‐7/del(7q), and had first undergone allo‐SCT between 2001 and 2018. Patients with recurrent cytogenetic abnormalities, including t(8;21), inv(16), t(16;16), t(15;17), t(9;22), inv(3), t(3;3), t(1;22), and 11q23 abnormalities, which are classified as distinct entities in the World Health Organization (WHO) classification of AML [11], were excluded. We also excluded cases missing data for the survival, relapse, and cytogenetic data at the diagnosis. This study was approved by the JDCHCT and by the institutional review board of Kanazawa University.

The primary endpoint was the overall survival (OS), and the secondary endpoints were the leukemia‐free survival (LFS), cumulative incidence of relapse (CIR), and nonrelapse mortality (NRM). The following karyotypic descriptions were considered as der(1;7)(q10;p10): +1, der(1;7)(q10;p10); der(1;7)(q10;p10); +1, der(1;7); der(7)t(1;7)(q11;p11), as previously reported [12]. Cases with chromosomal changes in addition to der(1;7) or ‐7/del(7q) were defined as having additional chromosomal abnormalities (ACAs).

The OS and LFS probabilities were estimated by the Kaplan–Meier method. The CIR and NRM were estimated using a cumulative incidence method, considering each risk as a competing risk. To analyze risk factors for transplant outcomes of AML patients with der(1;7), a multivariate analysis was performed using Cox‐proportional hazard tests for the OS and LFS and Fine‐Gray methods for the CIR and NRM. Variables with a *p* value of <0.10 in the univariate analyses and the presence of ACAs were included in the multivariate analysis using backward stepwise covariate selection. Two‐tailed *p* values of <0.05 were considered significant. All statistical analyses were performed with the EZR software program [13]. Additional information on methods is provided in the Supplemental Information.

## 2 RESULTS

### 2.1 Patient characteristics

Of the 16,241 patients with AML ≥16 years old who first underwent allo‐SCT between 2001 and 2018, a total of 151 AML patients with der(1;7) and 853 with ‐7/del(7q) were identified.

The baseline characteristics of each group are summarized in Table 1. The median follow‐up for surviving patients was 3.4 (range, 0.1–17.7) years. The median age at allo‐SCT was significantly older in the der(1;7) group than in the ‐7/del(7q) group (60 [range: 25–72] years old vs. 57 [16–82] years old, *p* < 0.001). The der(1;7) group was more male‐predominant than the ‐7/del(7q) group (88.7% vs. 68.3%, *p* < 0.001), and the WHO classification of AML subtypes differed significantly between the groups (*p* < 0.001). The presence of ACAs was similar between the groups (50.9% in the der(1;7) group vs. 57.4% in the ‐7/del(7q) group; *p* = 0.16), but patients with ≥2 ACAs were significantly rarer in the der(1;7) group than in the ‐7/del(7q) group (29.1% vs. 50.8%, *p* < 0.001). Del(20q) and +8 were common in the der(1;7) group, whereas ‐5/del(5q), which was common in the ‐7/del(7q) group, was rarely found in the der(1;7) group. The other variables did not differ markedly between the groups.

**TABLE 1:** Baseline characteristics of AML patients with der(1;7) (q10;p10) and those with ‐7/del(7q)

	der(1;7)(10;p10) (*N* = 151)	‐7/del(7q) (*N* = 853)	
Variables	*N* (%)	*N* (%)	*p*‐Value
Age at allo‐SCT, years (median, range)	60 (25–72)	57 (16–82)	<0.001
Age at allo‐SCT			
16–59 years	71 (47)	521 (65)	0.002
≥60 years	80 (53)	332 (35)	
Sex			
Male	134 (89)	583 (68)	<0.001
Female	17 (11)	270 (32)	
Year of allo‐SCT			
2001–2010	64 (42)	310 (36)	0.17
2011–2018	87 (58)	543 (64)	
Performance status			
0–1	120 (79)	681 (81)	0.74
2–4	31 (21)	164 (19)	
Not available	0	8	
HCT‐CI			
0–2	83 (63)	537 (73)	0.057
≥3	46 (35)	200 (27)	
Not available	22	116	
WBC count at the diagnosis			
<20,000	132 (87)	700 (85)	0.37
≥20,000	17 (11)	120 (15)	
Not available	2	33	
WHO classification			
AML with MRC	104 (68)	456 (55)	<0.001
Therapy‐related MNs	20 (13)	75 (9)	
AML NOS or other	26 (17)	299 (36)	
Not available	1	23	
Disease status at allo‐SCT			
CR1	39 (26)	214 (25)	0.91
CR ≥2	4 (3)	31 (4)	
Active disease	107 (71)	602 (71)	
Not available	1	6	
Conditioning			
MAC	90 (60)	520 (62)	0.65
RIC	61 (40)	321 (38)	
Not available	0	12	
Donor source			
Matched related	27 (18)	161 (19)	0.074
Mismatched related	6 (4)	83 (10)	
Matched unrelated	27 (18)	154 (18)	
Mismatched unrelated	31 (20)	123 (15)	
Umbilical cord blood	61 (40)	321 (38)	
Not available	1	11	
GVHD prophylaxis			
CsA based	46 (30)	252 (30)	0.29
TAC based	104 (70)	585 (69)	
other than CI	0 (0)	15 (2)	
Not available	1	1	
Number of ACAs			
0	74 (49)	366 (43)	<0.001
1	33 (22)	57 (7)	
≥2	44 (29)	433 (51)	
Variation of ACAs			
+8	31 (21)	87 (10)	<0.001
del (20q)	16 (11)	17 (2)	<0.001
‐5/del(5q)	2 (1)	221 (26)	<0.001
Others	53 (35)	477 (56)	<0.001
Time from diagnosis to allo‐SCT			
<6 months	83 (55)	518 (61)	0.18
≥6 months	68 (45)	334 (39)	
Not available	0	1	
Median follow‐up of survivors, years	3.4 (0.2–13.3)	3.4 (0.1–17.7)	0.75

Abbreviations: ACAs, additional chromosomal abnormalities; allo‐SCT, allogeneic hematopoietic stem cell transplantation; AML, acute myeloid leukemia; CI, calcineurin inhibitor; CR, complete remission; CsA, cyclosporine A; GVHD, graft‐versus‐host disease; HCT‐CI, hematopoietic cell transplantation comorbidity index; MAC, myeloablative conditioning; MNs, myeloid neoplasms; MRC, myelodysplasia‐related changes; NOS, not otherwise specified; RIC, reduced‐intensity conditioning; TAC, tacrolimus; WBC, white blood cell.

### 2.2 Transplant outcomes in each group

**FIGURE 1:**
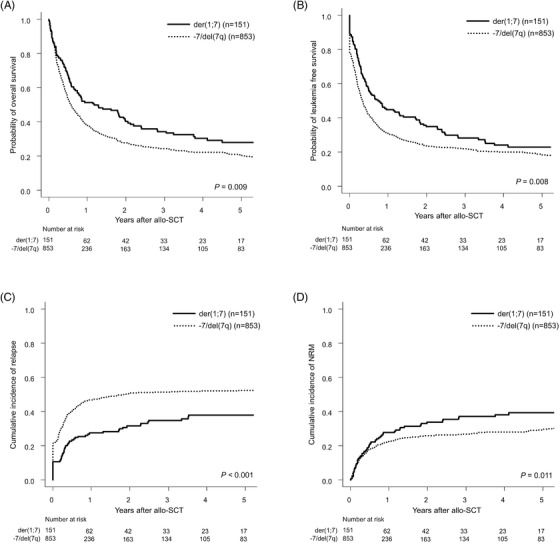
Outcome after allogeneic hematopoietic stem cell transplantation (allo‐SCT) in acute myeloid leukemia (AML) patients with der(1;7) (q10;p10) and those with ‐7/del(7q). The overall survival (OS) (A), leukemia‐free survival (LFS) (B), cumulative incidence of relapse (CIR) (C), and nonrelapse mortality (NRM) (D)

The 3‐year OS probability was significantly higher in the der(1;7) group than in the ‐7/del(7q) group (34.2% vs. 24.2%, *p* = 0.009) (Figure 1A). Similarly, the 3‐year LFS probability was significantly higher in the der(1;7) group than in the ‐7/del(7q) group (28.1% vs. 21.9%, *p* = 0.008) (Figure 1B). The 3‐year CIR was significantly lower in the der(1;7) group than in the ‐7/del(7q) group (34.8% vs. 51.3%, *p* < 0.001, Figure 1C). The 3‐year cumulative incidence of NRM was higher in the der(1;7) group than in the ‐7/del(7q) group (37.1% vs. 26.9%, *p* = 0.011, Figure 1). There were no significant differences between the groups in the cumulative incidences of acute or chronic graft‐versus‐host disease (Figure S1).

### 2.3 Risk factors for transplant outcome among der(1;7) patients

Univariate and multivariate analyses were performed for the der(1;7) patients to identify risk factors for transplant outcomes. The 3‐year OS of the patients without ACAs (*n* = 74) was 44.9%, which was similar to the OS in those with 1 ACA (*n* = 33, 31.3%, *p* = 0.11) but significantly higher than in those with ≥2 ACAs (*n* = 44; 18.6%, *p* < 0.001) (Figure S2, Table S1). A multivariate analysis demonstrated that the presence of ACAs was a significant risk factor for the OS (≥2 ACAs, hazard ratio [HR] 2.03 [95% confidence interval, CI: 1.25–3.28], *p* = 0.004), LFS (≥2 ACAs, HR 2.05 [95% CI: 1.30–3.22], *p* = 0.002). A poor performance status (PS) was also a significant risk factor for the OS (PS 2–4, HR 1.85 [95% CI: 1.12–3.05], *p* = 0.016), LFS (HR 1.87 [95% CI: 1.19–2.94], *p* = 0.007) and NRM (HR 2.21 [95% CI: 1.21–4.04], *p* = 0.010). Active disease at allo‐SCT was a significant risk factor for the CIR (HR 2.25 [95% CI: 1.13–4.45], *p* = 0.020) (Table S2).

## 3 DISCUSSION

Since der(1;7)(q10;p10) is a rare chromosomal abnormality in myeloid malignancies, der(1;7) could not be considered a distinct abnormality, and its clinical characteristics and the transplant outcome of AML patients with der(1;7) have not been fully clarified.

In this study, we identified some differences between AML patients with der(1;7) and those with ‐7/del(7q). AML patients with der(1;7) tended to be male and have a history of MDS or chemoradiotherapy, which was consistent with previous studies on MDS patients with der(1;7) [4, 5, 8]. The cytogenetic profiles also differed among these groups depending on the number and the variations of ACAs.

Previous studies have described the specific mutational profile in myeloid neoplasms with der(1;7). In those studies, der(1;7) patients had more somatic *RUNX1* and *ETNK1* mutations and fewer *TP53* mutations than ‐7/del(7q) patients (7, 8). As such, der(1;7) should be considered a distinct entity among myeloid malignancies.

Recent studies have suggested that der(1;7) patients with myeloid neoplasms may have a favorable prognosis. Ganster et al. compared MDS patients with isolated der(1;7) to those with isolated ‐7/del(7q) and showed that der(1;7) patients had a significantly better OS than those with monosomy 7 (median OS: 26 vs. 14 months), whereas patients with del(7q) had a similar OS (median OS: 44 vs. 26 months) [5]. Itonaga et al. reported that MDS patients with isolated der(1;7) had better transplant outcomes than those with isolated ‐7/del(7q), regardless of disease status [14]. In our study, AML patients with der(1;7) had a better OS and LFS than those with ‐7/del(7q), a trend that was similar to previous studies on MDS patients with der(1;7).

It was interesting to note that AML patients with der(1;7) had a higher NRM than those with ‐7/del(7q) in this study. This may have been partly because der(1;7) patients were older and tended to have higher hematopoietic cell transplantation comorbidity index scores, compared to ‐7/del(7q) patients.

Some studies showed that the presence of ACAs in MDS patients with der(1;7) did not significantly influence clinical outcomes [4, 15]. However, in our study, the presence of ACAs was identified as a significant risk factor for the OS and LFS in a multivariate analysis. These findings suggest that complication with ACAs may more strongly influence the prognosis of AML patients with der(1;7) than MDS patients with der(1;7).

This study has several limitations that should be noted. First, since this study was retrospective and only included patients who underwent allo‐SCT, there may have been patient selection bias, and it may not have reflected the actual prognosis of AML patients with der(1;7). Second, since we could not determine genetic aberrations due to a lack of data in TRUMP, the prognostic relevance of somatic mutations also remains unclear. Third, the number of patients was limited because of the rarity of this entity. Nevertheless, this nationwide study, to our knowledge, included the largest number of AML patients with der(1;7) to date.

In conclusion, our study demonstrated for the first time the transplant outcomes of AML patients with der(1;7). Although allo‐SCT is a feasible treatment option for AML patients with der(1;7), AML patients with der(1;7) and other ACAs have a poor outcome, even after allo‐SCT, so more efficient treatment strategies are required for this subgroup of patients.

## AUTHOR CONTRIBUTIONS

Hiroki Mizumaki and Ken Ishiyama designed the research, organized the project, analyzed data, and wrote the manuscript. Hiroki Mizumaki, Ken Ishiyama, Jun Aoki, Jinichi Mori, Shohei Mizuno, Noriko Doki, Takahiro Fukuda, Naoyuki Uchida, Masahito Onizuka, Masatsugu Tanaka, Yuta Katayama, Yukiyasu Ozawa, Kazuhiro Ikegame, Satoru Takada, Toshiro Kawakita, Nobuyuki Aotsuka, Yoshiko Atsuta, and Masamitsu Yanada critically reviewed the manuscript and checked the final manuscript.

## CONFLICT OF INTEREST

The authors declare no competing financial interests.

## FUNDING INFORMATION

The authors received no specific funding for this study.

## ETHICS STATEMENT

This study was approved by the JDCHCT (approval number: 2–48) and by the institutional review board of Kanazawa University (approval number: 2017–260).

## Supporting information

Supporting Information

## Data Availability

The data that support the findings of this study are available from the corresponding author upon reasonable request.
