# Supplementary material for: Outcomes after allogeneic hematopoietic stem cell transplantation in acute myeloid leukemia patients with der(1;7)(q10;p10)
Source: EJHaem. 2022 Nov 6;4(1):251–7. doi: 10.1002/jha2.609 (PMC9928652; doi:10.1002/jha2.609)
Supplement: Supplementary file 1 — Supporting Information [file JHA2-4-251-s001.docx]

**Supplemental information**

**Outcomes after allogeneic hematopoietic stem cell transplantation in patients with acute myeloid leukemia with der(1;7)(q10;p10)**

Hiroki Mizumaki, Ken Ishiyama, Jun Aoki, Jinichi Mori, Shohei Mizuno, Noriko Doki, Takahiro Fukuda, Naoyuki Uchida, Masahito Onizuka, Masatsugu Tanaka, Yuta Katayama, Yukiyasu Ozawa, Kazuhiro Ikegame, Satoru Takada, Toshiro Kawakita, Nobuyuki Aotsuka, Yoshiko Atsuta and Masamitsu Yanada

**Supplemental Methods**

*Definition*

The overall survival (OS) was defined as the time between the date of allogeneic hematopoietic stem cell transplantation (allo-SCT) and death due to any cause or the last visit. The leukemia-free survival (LFS) was defined as the time interval from allo-SCT to either relapse or death, whichever came first. The non-relapse mortality (NRM) was defined as death without evidence of disease recurrence after allo-SCT. The performance status (PS) was evaluated according to the Eastern Cooperative Oncology Group (ECOG). Human leukocyte antigen (HLA)-matched related donors were serologic 6/6 HLA-A, HLA-B and HLA-DR matched donors, and HLA-matched unrelated donors were donors matched at the allele level of 8/8 HLA-A, HLA-B, HLA-C or HLA-DRB1 alleles. Conditioning intensity was classified as myeloablative-intensity conditioning (MAC) or reduced-intensity conditioning (RIC) according to the established criteria (1, 2). Acute and chronic graft-versus-host disease (GVHD) were assessed according to the established criteria (3, 4). For the chronic GVHD analysis, only patients who survived 100 days after allo-SCT without relapse were included.

*Study approval*

This retrospective study was performed in accordance with the Declaration of Helsinki. This study was designed by the Acute Myeloid Leukemia Working Group of the Japanese Society for Transplantation and Cellular Therapy (JSTCT) and approved by the Japanese Data Center for Hematopoietic Cell Transplantation (approval no. 2-48) and by the institutional review board of Kanazawa University (approval no. 2017-260).

*Statistical analyses*

We compared patient characteristics and transplant outcomes. Categorical variables were compared by Fisher’s exact test. Continuous variables were compared by the Mann-Whitney U test. The OS and LFS probabilities were estimated using the Kaplan-Meier method. A univariate analysis for the survival was performed using the log-rank test. The cumulative incidence of relapse (CIR) and NRM were evaluated using Gray’s method, considering each risk as a competing risk. Cumulative incidences of acute and chronic GVHD were also evaluated using Gray’s method, considering relapse and non-relapse death as competing risks. A multivariate analysis including significant variables from the univariate analysis was performed using Cox-proportional hazard tests for the OS and LFS and Fine-Gray methods for the CIR and NRM. Variables considered in univariate models for each analysis included those based on patient characteristics, i.e. the age at allo-SCT (16-59, ≥60 years old), sex (male, female), year at allo-SCT (2001-2010, 2011-2018), PS (0-1, 2-4), Hematopoietic Cell Transplant Comorbidity Index (HCT-CI) (0-2, ≥3), WBC count at the diagnosis (<20,000, ≥20,000), WHO classification of acute myeloid leukemia (AML) subtypes (therapy-related myeloid neoplasms, AML with myelodysplasia-related changes, AML not otherwise specified[NOS]), disease status at allo-SCT (complete remission [CR], active disease), conditioning regimen (MAC, RIC), donor source (HLA-matched related, HLA-mismatched related, HLA-matched unrelated, HLA-mismatched unrelated, umbilical cord blood), GVHD prophylaxis (cyclosporine-based, tacrolimus-based), number of additional chromosomal abnormalities (ACAs) (0, 1, ≥2 ACAs) and time from diagnosis to allo-SCT (<6 months, ≥6 months). In the analysis among patients with der(1;7), factors with at least borderline significance (p <0.10) in the univariate analyses and the presence of ACAs (0, 1, ≥2 ACAs) were included in a multivariate analysis using backward stepwise covariate selection. Two-tailed p-values of <0.05 were considered significant. Hazard ratios and 95% confidence intervals were also calculated. All statistical analyses were performed with EZR, a graphical user interface for R software (The R Foundation for Statistical Computing, version 4.1.2, Vienna, Austria) (5).

**References**

1. Giralt S, Ballen K, Rizzo D, Bacigalupo A, Horowitz M, Pasquini M, et al. Reduced-intensity conditioning regimen workshop: defining the dose spectrum. Report of a workshop convened by the center for international blood and marrow transplant research. Biol Blood Marrow Transplant. 2009;15(3):367-9.

2. Bacigalupo A, Ballen K, Rizzo D, Giralt S, Lazarus H, Ho V, et al. Defining the intensity of conditioning regimens: working definitions. Biol Blood Marrow Transplant. 2009;15(12):1628-33.

3. Przepiorka D, Weisdorf D, Martin P, Klingemann HG, Beatty P, Hows J, et al. 1994 Consensus Conference on Acute GVHD Grading. Bone Marrow Transplant. 1995;15(6):825-8.

4. Sullivan KM, Agura E, Anasetti C, Appelbaum F, Badger C, Bearman S, et al. Chronic graft-versus-host disease and other late complications of bone marrow transplantation. Semin Hematol. 1991;28(3):250-9.

5. Kanda Y. Investigation of the freely available easy-to-use software 'EZR' for medical statistics. Bone Marrow Transplant. 2013;48(3):452-8.

**Table S1.** **Results of a univariate analysis of the OS, LFS, CIR and NRM at three years in AML patients with der(1;7)(10;p10).**

|  | | Number | OS | | LFS | | CIR | | NRM | |
| --- | --- | --- | --- | --- | --- | --- | --- | --- | --- | --- |
|  |  |  | % (95% CI) | *P* *value* | % (95% CI) | *P* *value* | % (95% CI) | *P* *value* | % (95% CI) | *P* *value* |
| Age at allo-SCT | 16-59 years | 71 | 41 (29-52) | 0.21 | 33 (22-44) | 0.36 | 37 (25-48) | 0.71 | 31 (20-42) | 0.24 |
|  | ≥60 years | 80 | 28 (17-39) |  | 24 (14-35) |  | 33 (23-44) |  | 43 (32-54) |  |
| Sex | Male | 134 | 32 (24-41) | 0.97 | 28 (20-36) | 0.10 | 36 (28-44) | 0.17 | 38 (29-46) | 0.17 |
|  | Female | 17 | 51 (23-73) |  | 33 (13-56) |  | 26 (8-48) |  | 34 (12-58) |  |
| Year of allo-SCT | 2001-2010 | 64 | 33 (21-44) | 0.39 | 23 (13-34) | 0.45 | 40 (28-52) | 0.51 | 37 (25-49) | 0.99 |
|  | 2011-2018 | 87 | 35 (24-46) |  | 32 (22-43) |  | 30 (20-41) |  | 37 (27-48) |  |
| Performance status | 0-1 | 120 | 38 (28-47) | <0.001 | 32 (23-41) | <0.001 | 34 (25-42) | 0.73 | 34 (26-43) | 0.014 |
|  | 2-4 | 31 | 20 (8-36) |  | 13 (4-27) |  | 39 (22-55) |  | 48 (30-64) |  |
| WBC count at the diagnosis | <20,000 | 132 | 33 (24-41) | 0.58 | 28 (20-36) | 0.59 | 33 (25-42) | 0.33 | 39 (30-47) | 0.17 |
|  | ≥20,000 | 17 | 50 (24-71) |  | 33 (12-56) |  | 49 (24-70) |  | 18 (5-40) |  |
| HCT-CI | 0-2 | 83 | 30 (20-41) | 0.53 | 24 (15-35) | 0.63 | 41 (30-52) | 0.038 | 35 (24-45) | 0.015 |
|  | ≥3 | 46 | 32 (18-47) |  | 27 (14-41) |  | 22 (11-35) |  | 51 (35-65) |  |
| WHO classification of AML | AML with MRC | 104 | 31 (21-40) | 0.55 | 35 (16-55) | 0.39 | 37 (27-46) | 0.56 | 39 (29-48) | 0.50 |
|  | Therapy-related MNs | 20 | 43 (21-64) |  | 25 (16-34) |  | 25 (9-45) |  | 40 (19-60) |  |
|  | AML NOS | 26 | 45 (24-64) |  | 38 (19-57) |  | 36 (18-55) |  | 27 (12-45) |  |
| Disease status at allo-SCT | CR | 44 | 62 (39-79) | 0.033 | 44 (27-59) | 0.016 | 27 (14-42) | 0.048 | 39 (23-54) | 0.93 |
|  | Active disease | 107 | 52 (40-63) |  | 26 (18-34) |  | 38 (29-48) |  | 36 (27-45) |  |
| Conditioning | MAC | 90 | 35 (25-45) | 0.78 | 31 (21-42) | 0.37 | 32 (22-42) | 0.34 | 37 (27-47) | 0.93 |
|  | RIC | 61 | 33 (21-46) |  | 23 (13-35) |  | 39 (27-51) |  | 37 (25-50) |  |
| Donor source | Matched related | 27 | 56 (34-73) | 0.072 | 35 (17-53) | 0.39 | 49 (28-67) | 0.67 | 16 (5-33) | 0.13 |
|  | Mismatched related | 6 | 17 (8-52) |  | 34 (15-54) |  | 50 (11-80) |  | 33 (5-68) |  |
|  | Matched unrelated | 27 | 33 (14-53) |  | 17 (8-52) |  | 26 (10-44) |  | 41 (20-60) |  |
|  | Mismatched unrelated | 31 | 25 (11-42) |  | 18 (7-33) |  | 33 (17-49) |  | 50 (31-66) |  |
|  | Umbilical cord blood | 61 | 33 (21-46) |  | 32 (20-44) |  | 31 (20-43) |  | 37 (25-49) |  |
| GVHD prophylaxis | CsA based | 46 | 34 (21-48) | 0.40 | 22 (11-36) | 0.16 | 44 (29-58) | 0.26 | 34 (21-48) | 0.57 |
|  | TAC based | 104 | 34 (25-44) |  | 31 (22-41) |  | 30 (21-39) |  | 39 (29-49) |  |
| Number of ACAs | 0 | 74 | 47 (34-58) | <0.001 | 40 (28-51) | <0.001 | 31 (20-42) | 0.18 | 30 (20-41) | 0.14 |
|  | 1 | 33 | 31 (15-49) |  | 24 (9-41) |  | 30 (15-48) |  | 46 (27-63) |  |
|  | ≥2 | 44 | 19 (8-32) |  | 13 (5-25) |  | 44 (29-58) |  | 43 (29-57) |  |
| Time from diagnosis to  allo-SCT | <6 months | 83 | 37 (26-48) | 0.97 | 30 (20-40) | 0.74 | 33 (23-43) | 0.69 | 38 (27-48) | 0.46 |
|  | ≥6 months | 68 | 30 (19-43) |  | 26 (15-38) |  | 38 (26-50) |  | 36 (25-48) |  |

Abbreviations: AML, acute myeloid leukemia; OS, overall survival; LFS, leukemia-free survival; CIR, cumulative incidence of relapse; NRM, non-relapse mortality; allo-SCT, allogeneic hematopoietic stem cell transplantation; WBC, white blood cell; HCT-CI, hematopoietic cell transplantation comorbidity index; MRC, myelodysplasia-related changes; MNs, myeloid neoplasms; NOS, not otherwise specified; CR; complete remission; MAC, myeloablative conditioning; RIC, reduced-intensity conditioning; CsA, cyclosporine; TAC, tacrolimus; ACAs, additional chromosomal abnormalities

**Table S2. Results of a multivariate analysis of the OS, LFS, CIR, and NRM in AML patients with der(1;7)(10;p10).**

| Outcomes | Variables | | HR (95% CI) | *P* *value* |
| --- | --- | --- | --- | --- |
| OS | Performance status | 0-1 | 1.00 |  |
|  |  | 2-4 | 1.85 (1.12-3.05) | 0.016 |
|  | Number of ACAs | 0 | 1.00 |  |
|  |  | 1 | 1.69 (0.98-2.90) | 0.059 |
|  |  | ≥2 | 2.03 (1.25-3.28) | 0.004 |
| LFS | Performance status | 0-1 | 1.00 |  |
|  |  | 2-4 | 1.87 (1.19-2.94) | 0.007 |
|  | Number of ACAs | 0 | 1.00 |  |
|  |  | 1 | 1.54 (0.94-2.52) | 0.086 |
|  |  | ≥2 | 2.05 (1.30-3.22) | 0.002 |
| CIR | Disease status at allo-SCT | CR | 1 |  |
|  |  | Active disease | 2.25 (1.13-4.45) | 0.020 |
|  | HCT-CI | 0-2 | 1 |  |
|  |  | ≥3 | 0.43 (0.21-0.89) | 0.024 |
| NRM | Performance status | 0-1 | 1 |  |
|  |  | 2-4 | 2.21 (1.21-4.04) | 0.010 |

Abbreviations: HR; hazard ratio; OS, overall survival; LFS, leukemia-free survival; CIR, cumulative incidence of relapse; NRM, non-relapse mortality; ACAs, additional chromosomal abnormalities; CR, complete remission; allo-SCT, allogeneic hematopoietic stem cell transplantation; HCT-CI, hematopoietic cell transplantation comorbidity index.

**
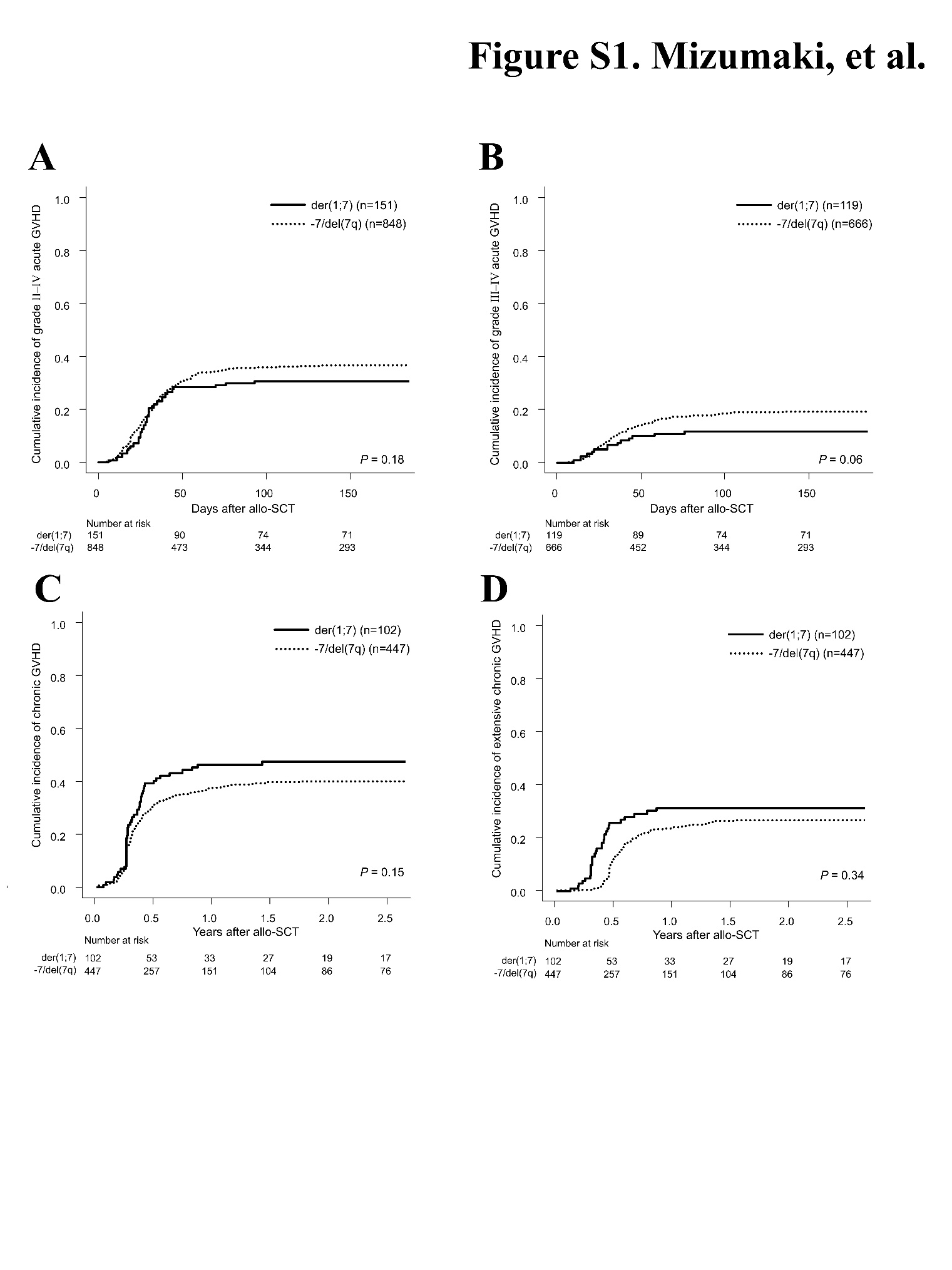
**

**Figure S1.** Cumulative incidences of grade II–IV acute graft-versus-host disease (GVHD) (A), grade III–IV acute GVHD (B), chronic GVHD (C) and extensive chronic GVHD (D) in AML patients with der(1;7) and -7/del(7q). The 100-day cumulative incidences of grade II–IV and III–IV acute GVHD were 30.4% (95% confidential interval [CI]: 23.3%-37.9%) and 11.2% (95% CI: 6.8%-18.3%) in the der(1;7) group and 36.0% (95% CI: 32.8%-39.2%) and 18.8% (95% CI: 15.9%-21.9%) in the -7/del(7q) group, respectively. The 1-year cumulative incidences of chronic and extensive chronic GVHD were 46.3% (95% CI: 36.3%-55.6%) and 31.4% (95% CI: 22.4%-40.8%) in the der(1;7) group and 37.2% (95% CI: 32.7%-41.8%) and 35.1% (95% CI: 21.1%-29.3%) in the -7/del(7q) group, respectively.

**
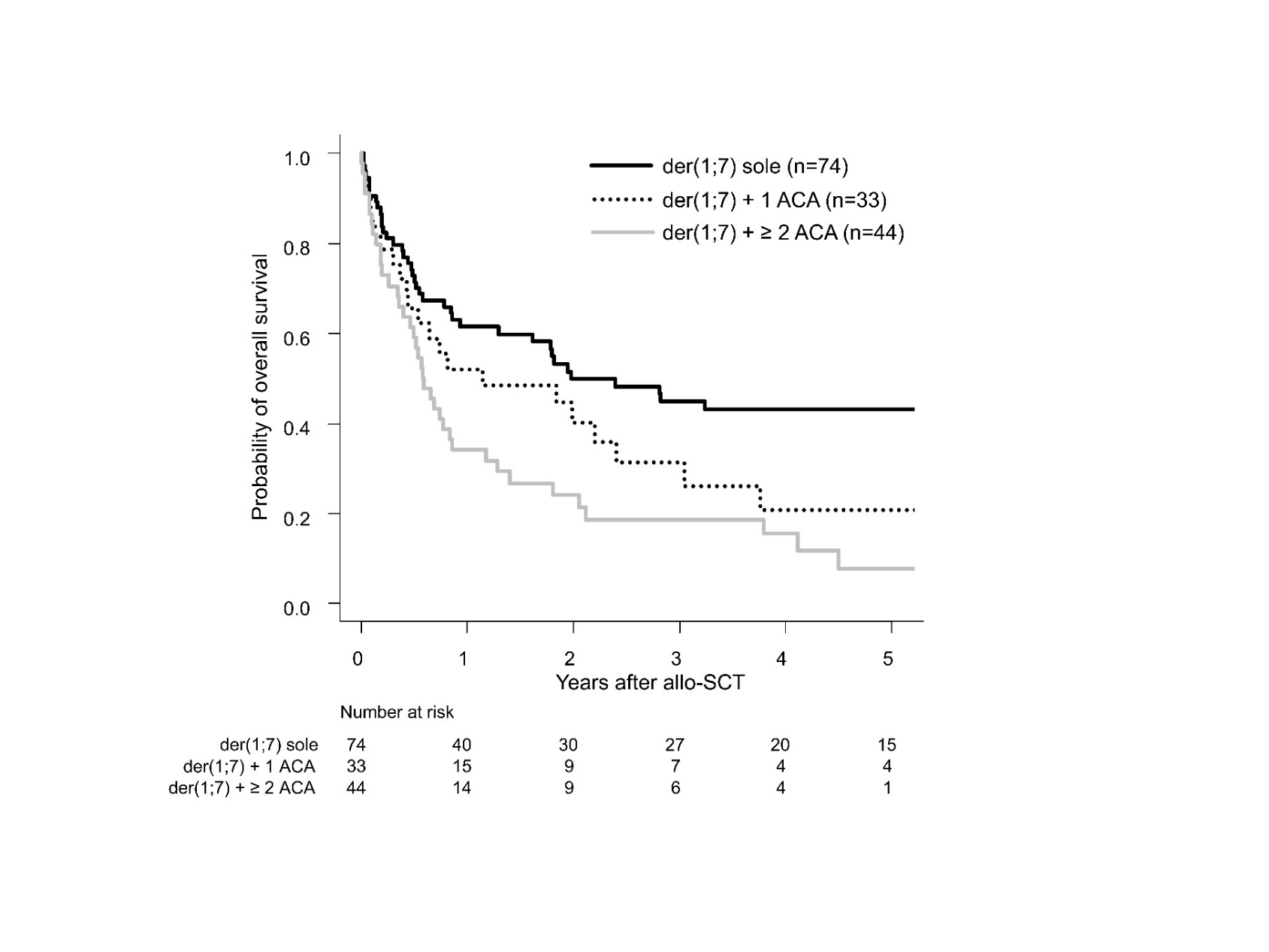
**

**Figure S2.** The probabilities of the overall survival (OS) after allo-SCT for AML patients with der(1;7)(q10;p10) according to the number of additional chromosomal abnormalities (ACAs). The 3-year OS was 44.9% (95% CI: 32.7%-56.4%) in the patients without ACA, 31.3% (95% CI: 15.0%-49.1%) in the patients with 1 ACA, and 18.6% (95% CI: 8.5%-31.7%) in the patients with ≥2 ACAs.
